# Supplementary material for: Duodenojejunal Omega Switch Surgery Reduces Oxidative Stress Induced by Cafeteria Diet in Sprague-Dawley Rats
Source: Nutrients. 2022 Oct 2;14(19):4097. doi: 10.3390/nu14194097 (PMC9573765; doi:10.3390/nu14194097)
Supplement: Supplementary file 1 [file nutrients-14-04097-s001.zip › nutrients-1946963-supplementary.pdf]

Supplementary Materials

Duodenojejunal Omega Switch Surgery Reduces Oxidative Stress Induced by Cafeteria Diet in Sprague-Dawley Rats

Jakub Poloczek [1,2], Wojciech Kazura [3], Elżbieta Chełmecka [4], Katarzyna Michalczyk [3], Jerzy Jochem [3], Janusz Gumprecht [2] and Dominika Stygar [3,*]

Table S1. Levels of antioxidant and lipid peroxidation markers in the plasma of Sprague-Dawley rats subjected to duodenojejunal omega switch (DJOS) and control (SHAM) surgery and different dietary protocols at 8 weeks after the surgery. The data are presented as median and interquartile range, Me (Q1-Q3).

Parameter	Surgery	Dietary protocols			
		CD/CD	CD/HFS	HFS/CD	HFS/HFS
SOD activity [NU/mg]	DJOS	22.4 (22.3-22.4)	24.8 (24.6-24.9)	22.3 (22.8-23.5)	20.5 (20.3-20.6)
	SHAM	29.4 (29.1-29.7)	25.9 (25.3-26.5)	25.9 (25.4-26.1)	28.2 (27.7-28.5)
CER activity [mg/dL]	DJOS	22.4 (22.2-22.5)	45.4 (43.7-46.6)	30.5 (30.4-30.8)	25.6 (25.5-25.7)
	SHAM	25.6 (25.3-26.8)	48.4 (48.2-48.5)	34.7 (34.6-35.0)	55.5 (55.3-55.7)
TAC level [mmol/L]	DJOS	0.86 (0.86-0.88)	0.51 (0.51-0.52)	0.57 (0.57-0.59)	0.65 (0.65-0.65)
	SHAM	0.86 (0.84-0.87)	0.37 (0.31-0.39)	0.46 (0.43-0.49)	0.28 (0.27-0.29)
MDA concentration [µmol/L]]	DJOS	2.3 (2.2-2.4)	4.8 (4.7-4.9)	4.2 (4.2-4.3)	3.6 (3.4-3.8)
	SHAM	2.7 (2.5-2.8)	5.5 (5.2-5.6)	5.5 (5.3-5.7)	3.5 (3.4-3.6)
LS concentration [RF]	DJOS	33.5 (32.6-36.0)	76.4 (76.3-76.6)	58.1 (58.0-58.5)	75.4 (75.2-75.7)
	SHAM	54.7 (54.2-54.9)	99.4 (99.3-99.5)	82.3 (82.1-82.4)	96.8 (96.3-98.2)

Legend: CD – control diet, CER – cerulopasmin, DJOS – duodenojejunal omega switch surgery, HFS – high fat/high sugar diet, LS – lipofuscin, MDA – malondialdehyde, NU – nitrate units, RF – relative lipid extract fluorescence, SHAM – control surgery, SOD – superoxide dismutase, TAC – total antioxidant capacity.

Table S2. The significance level for multiple comparisons in non-parametric Kruskal-Wallis analysis of antioxidant and lipid peroxidation markers in the plasma of Sprague-Dawley rats subjected to duodenojejunal omega switch (DJOS) and control (SHAM) surgery and different dietary protocols at 8 weeks after the surgery.

**Parameter**	**Surgery**	$p_{CD/CD}$ **vs** $p_{CD/HFS}$	$p_{CD/CD}$ **vs** $p_{HFS/CD}$	$p_{CD/CD}$ **vs** $p_{HFS/HFS}$	$p_{CD/HFS}$ **vs** $p_{HFS/CD}$	$p_{CD/HFS}$ **vs** $p_{HFS/CF}$	$p_{HFS/CD}$ **vs** $p_{HFS/HFS}$
SOD	DJOS	< 0.05	1	0.724	0.724	< 0.001	< 0.05
	SHAM	< 0.01	< 0.01	0.849	1	0.164	0.164
CER	DJOS	< 0.001	< 0.05	0.849	0.849	< 0.05	0.849
	SHAM	< 0.01	0.849	< 0.001	0.849	0.849	< 0.05
TAC	DJOS	< 0.001	< 0.05	0.849	0.849	< 0.05	0.849
	SHAM	< 0.05	0.849	< 0.001	0.849	0.849	< 0.05
MDA	DJOS	< 0.001	< 0.05	0.849	1	< 0.05	0.724
	SHAM	< 0.01	< 0.01	0.849	1	0.202	0.133
LS	DJOS	< 0.001	0.849	< 0.05	< 0.05	0.849	0.849
	SHAM	< 0.001	0.849	< 0.05	< 0.05	0.990	0.785

Legend: CD – control diet, CD/CD – rats fed with CD before and after the surgery, CD/HFS – rats fed with CD before and HFS diet after the surgery, CER – ceruloplasmin activity, DJOS – duodenojejunal omega switch surgery, HFS – high fat/high sugar diet, HFS/CD – rats fed with HFS diet before and CD after the surgery, HFS/HFS – rats fed with HFS diet before and after the surgery, LS – lipofuscin concentration, MDA – malondialdehyde concentration, SHAM – control surgery, SOD – superoxide dismutase activity, TAC – total antioxidant capacity.
